# Germline landscape of *RPA1, RPA2 and RPA3* variants in pediatric malignancies: identification of *RPA1* as a novel cancer predisposition candidate gene

**DOI:** 10.3389/fonc.2023.1229507

**Published:** 2023-10-06

**Authors:** Richa Sharma, Ninad Oak, Wenan Chen, Rose Gogal, Martin Kirschner, Fabian Beier, Michael J. Schnieders, Maria Spies, Kim E. Nichols, Marcin Wlodarski

**Affiliations:** ^1^ Department of Hematology, St. Jude Children´s Research Hospital, Memphis, TN, United States; ^2^ Department of Oncology, St. Jude Children’s Research Hospital, Memphis, TN, United States; ^3^ Center for Applied Bioinformatics, St. Jude Children’s Research Hospital, Memphis, TN, United States; ^4^ Department of Biochemistry, Carver College of Medicine, University of Iowa, Iowa City, IA, United States; ^5^ Department of Hematology, Oncology, Hemostaseology and Stem Cell Transplantation, Medical Faculty, RWTH Aachen University, Aachen, Germany; ^6^ Center for Integrated Oncology Aachen Bonn Cologne Düsseldorf (CIO ABCD), Bonn, Germany

**Keywords:** RPA1, RPA2, RPA3, germline mutation, cancer

## Abstract

Replication Protein A (RPA) is single-strand DNA binding protein that plays a key role in the replication and repair of DNA. RPA is a heterotrimer made of 3 subunits – RPA1, RPA2, and RPA3. Germline pathogenic variants affecting *RPA1* were recently described in patients with Telomere Biology Disorders (TBD), also known as dyskeratosis congenita or short telomere syndrome. Premature telomere shortening is a hallmark of TBD and results in bone marrow failure and predisposition to hematologic malignancies. Building on the finding that somatic mutations in RPA subunit genes occur in ~1% of cancers, we hypothesized that germline RPA alterations might be enriched in human cancers. Because germline *RPA1* mutations are linked to early onset TBD with predisposition to myelodysplastic syndromes, we interrogated pediatric cancer cohorts to define the prevalence and spectrum of rare/novel and putative damaging germline *RPA1*, *RPA2*, and *RPA3* variants. In this study of 5,993 children with cancer, 75 (1.25%) harbored heterozygous rare (non-cancer population allele frequency (AF) < 0.1%) variants in the RPA heterotrimer genes, of which 51 cases (0.85%) had ultra-rare (AF < 0.005%) or novel variants. Compared with Genome Aggregation Database (gnomAD) non-cancer controls, there was significant enrichment of ultra-rare and novel *RPA1*, but not *RPA2* or *RPA3*, germline variants in our cohort (adjusted p-value < 0.05). Taken together, these findings suggest that germline putative damaging variants affecting *RPA1* are found in excess in children with cancer, warranting further investigation into the functional role of these variants in oncogenesis.

## Introduction

Maintenance of genome integrity requires efficient DNA repair. The perturbation of processes engaged in repair of DNA damage by somatic mutations is a well-known mechanism for oncogenesis. Germline biallelic inactivation of genes governing DNA repair leads to classic cancer predisposition syndromes such as Fanconi anemia, ataxia telangiectasia and Bloom syndrome, among others (1–4). Monoallelic mutations impacting some of these genes can also increase the risk for cancer (5–9). We recently discovered that germline heterozygous mutations in the Replication Protein A1 (*RPA1*) gene cause Telomere Biology Disorder (TBD), a hereditary condition classically associated with pathological shortening of telomeres resulting in bone marrow failure (BMF), pulmonary and liver fibrosis, mucocutaneous fragility, and predisposition to solid tumors, myelodysplastic syndromes (MDS) and acute myeloid leukemia (AML) (10).

The RPA1 protein is the largest subunit of Replication Protein A (RPA), a heterotrimeric complex consisting of RPA1 (RPA70), RPA2 (RPA32) and RPA3 (RPA14). As a complex, RPA tightly binds single-strand DNA (ssDNA) to protect it from nucleases while maintaining DNA accessible to essential DNA-DNA and DNA-protein interactions. Consistent with the ubiquitous and ongoing formation of ssDNA, RPA is present and required across almost all cellular processes during replication, recombination, and repair of DNA. In fact, RPA is involved in all ssDNA repair pathways (nucleotide excision, base excision, mismatch) and double strand DNA repair mechanisms (homologous recombination, non-homologous end joining) (11–13). RPA participates in such diverse pathways through its ability to dynamically bind ssDNA while facilitating DNA repair and cell cycle protein interactions (11).

The essential role of RPA in DNA repair might lend RPA to be mutated in cancers. By mining the Catalogue Of Somatic Mutations In Cancer (COSMIC) database (14), we found that somatic mutations in *RPA1*, *RPA2*, *RPA3* are found in 1.4%, 0.5%, and 0.9% of human cancers, respectively. In our previously published cohort of 4 patients with TBD, one patient who carried a germline *RPA1* p.V227A mutation developed advanced MDS requiring hematopoietic stem cell transplantation. All 3 *RPA1* germline mutations (p.V227A, p.E240K, p.T270A) identified in the 4 cases were missense and 2 out of 3 exerted a gain-of-function effect, resulting in increased binding to single strand and telomeric DNA (10). Besides these descriptions associating germline *RPA1* variants with bone marrow failure or hematologic malignancies, the *RPA2* or *RPA3* genes have not been linked to any human diseases thus far. Moreover, the landscape of germline variants in RPA heterotrimer in malignancies has not been systematically assessed. To address this knowledge gap, we investigated the occurrence of novel and rare germline variants in *RPA1*, *RPA2* and *RPA3* genes, in a cohort of 5,993 children with cancers. We found that ultra-rare and novel germline variants in the *RPA1* gene were significantly more common among pediatric cancer patients than non-cancer controls. Furthermore, we examined a separate cohort of 41 young adults with AML and identified potentially deleterious *RPA1* germline variants in 3 cases. Our studies indicate that the *RPA1* gene may be a novel risk factor for malignancies.

## Methods

### Data sources

For this study, we used publicly available whole exome sequencing datasets previously collected across studies at St. Jude Children’s Research Hospital or through dbGaP. Specifically, we used the Pediatric Cancer Genome Project (PCGP) (15), real-time clinical genomics (RTCG/G4K) (16), St. Jude Lifetime Cohort (SJLIFE) (17), and TARGET datasets (TARGET URL is https://www.ncbi.nlm.nih.gov/projects/gap/cgi-bin/study.cgi?study_id=phs000218). In sum, we interrogated 5,993 germline samples across 24 cancer types including hematologic, non-central nervous system (CNS) solid tumors, and CNS tumors. Cancers were stratified into hematologic (n = 3,452; 58%), solid (n = 1,974; 33%) or CNS (n = 1,068; 18%) cancers and further subclassified as follows: i) hematologic malignancies: B-cell (B) acute lymphoid leukemia (B-ALL), T-cell ALL (T-ALL), acute myeloid leukemia (AML), Hodgkin’s lymphoma, and non-Hodgkin’s lymphoma; ii) solid tumors: germ cell tumor (GCT), melanoma (MEL), neuroblastoma (NBL), nasopharyngeal carcinoma (NPC), papillary thyroid carcinoma (PTC), sarcomas (Ewing’s (EWS), osteosarcoma (OS), rhabdomyosarcoma (RMS), synovial), Wilms tumor (WT); and iii) CNS tumors: ependymoma (EP), low grade glioma (LGG), medulloblastoma (MB), and high grade glioma (HGG). An external cohort was queried, which consisted of 41 patients with AML from the German Study Alliance Leukemia that met following criteria: age below 35, blast-free remission after chemotherapy, karyotype aberrations (n = 12 with < 3, n = 29 with ≥ 3 aberrations detected in diagnostic karyotype or FISH analysis), and samples of peripheral blood or bone marrow at remission (18). The current study was approved by the Institutional Review Board at St. Jude Children’s Research Hospital.

### Variant calling and filtering

Variant calling and genotyping were performed using Genome Analysis Toolkit’s (GATK) best practices workflow with modifications as described previously (19). We retained high quality variants that passed filtering using following criteria: allelic balance > 0.2, genotype quality > 20, variant allelic frequency (VAF) for heterozygous variants between 20-80%, minimum of 10 alternate reads supporting single nucleotide variants (SNVs) and 7 alternate reads supporting InDels, and missingness < 25% of samples. We performed variant annotation using ANNOtate VARiation (ANNOVAR) and variant effect predictor (VEP) tools (20). We also annotated all the variants using InterVar (21) automated clinical interpretation based on the American College of Medical Genetics and Genomics (ACMG) guidelines (22).

We retained coding variants in the RPA heterotrimer (*RPA1*, *RPA2*, and *RPA3* genes) with genome aggregation database (gnomAD) non-cancer cohort allelic frequency (AF) of < 0.5% (23) of the following classes: missense, frameshift insertions and deletions, stop gain, and splice site. We further filtered to retain missense variants with a computed Combined Annotation-Dependent Depletion (CADD) (24) Phred score > 15.

### Computational analysis of RPA mutations

We performed local coordinate minimization followed by global side-chain optimization with the Atomic Multipole Optimized Energetics for Biomolecular Applications (AMOEBA) polarizable force field (25) on 5 high resolution structures of RPA fragments collectively comprising 7 modular domain of RPA heterotrimer. These included X-ray structures of the DNA binding domains A and B, DBD-A and DBD-B (PDB: 1JMC) (26), and the RPA trimerization core composed of DBD-C, D and E (PDB: 1L1O) (27) and NMR structures of the DBD-F (5N8A) (28) and the wing helix domain (PDB: 1DPU) (29). Prior to minimization, the ssDNA was removed from the 1JMC structure and bound peptides were removed from the 2 NMR structures. We then used our optimized structures to predict protein stability differences ΔΔG_Fold_ (DDG untrained (DDGun)) (30). DDGun estimates the ΔΔG_Fold_ of missense variants from a linear regression of sequence and biochemical features determined from the protein structure. Destabilizing ΔΔG_Fold_ values indicate a decrease in the ratio of folded to unfolded protein due to the mutation (we define negative ΔΔG_Fold_ values as stabilizing and positive ΔΔG_Fold_ values as destabilizing). We established ΔΔG_Fold_ cut-offs for mutations highly likely to impact protein folding. Our cut-offs were determined based on a ΔΔG_Fold_ that affects the ratio of folded to unfolded protein 12-fold (~1.5 kcal/mol) for both stabilizing and destabilizing mutations.

### Statistical analysis

We performed rare-variant burden tests for *RPA1*, *RPA2*, *RPA3* variants using 5,993 cases from all pediatric cancers in our cohorts (pan-cancer) and within each sub-class of cancers, namely, hematologic (n = 3,452), solid (n = 1,974), and central nervous system CNS (n = 1,068) malignancies. For the control set, we retrieved all variants across *RPA1, RPA2*, and *RPA3* from gnomAD v2 non-cancer subset containing 134,187 individuals with no reported malignancy (23). All variants from control dataset were processed through the same variant annotation and filtering workflow as our cancer cohort (AF < 0.5%). Enrichment tests for cases with and without germline ultra-rare (AF < 0.005%) plus novel (AF 0%) and rare (AF < 0.1%) variants in the 3 genes were performed using both two- and one-sided Fisher exact tests using the statistical package R (v4.3) described in previous studies (31, 32). We used Bonferroni correction to adjust for multiple testing with a significance cutoff of adjusted p-value of < 0.05.

## Results

### Variants identified among the RPA heterotrimer genes

**Figure 1:**
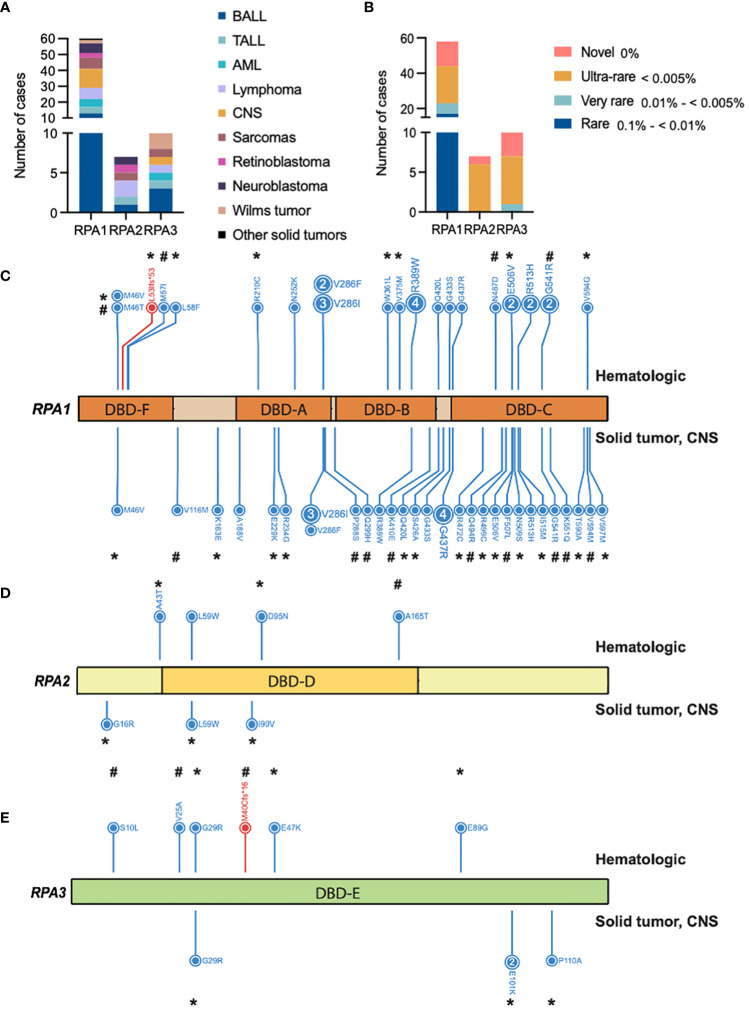
Germline heterozygous variants in the RPA heterotrimer in pediatric cancers. **(A)** Number of pediatric cancer cases with either RPA1, RPA2 or RPA3 heterozygous germline variants. B-ALL (B cell acute lymphoid leukemia), T-ALL (T cell acute lymphoid leukemia), AML (acute myeloid leukemia). Unique cancers are identified by different colors represented in the legend. **(B)** Number of cancer cases with either novel (pink), ultra-rare (gold), very rare (light teal), or rare (blue) germline variants in RPA1, RPA2 or RPA3 according to gnomAD allelic frequency. Schematic of human RPA1 (DNA binding domain (DBD- F, A, B, C)) **(C)**, RPA2 (DBD-D) **(D)** and RPA3 (DBD-E) **(E)** proteins with germline variants denoted. Blue and red lettering represents missense and frameshift variants, respectively. Numbers within circles represent the number of cases that harbored that variant while lack of numbering denotes one case per variant. Variants found in hematologic cases are represented on top and solid (intra and extra cranial) malignancies are denoted at the bottom of each protein map. * = ultra-rare variant allelic frequency (< 0.005%), # = novel variants.

Within the pan-cancer cohort, we identified 80 cases with 55 germline heterozygous *RPA1*, *RPA2* or *RPA3* variants meeting criteria of AF < 0.5% in gnomAD non-cancer cohort and CADD score > 15 for candidate variant selection (**Figure 1A**). Specifically, 40 *RPA1*, 7 *RPA2* and 8 *RPA3* unique heterozygous germline variants were identified in 63, 7 and 10 cases, respectively (**Figure 1B**). All variants were classified as variant of uncertain significance (VUS) according to the ACMG criteria (**Tables 1**–**3**). Majority of the variants (92% of *RPA1*, 71% of *RPA2* and all *RPA3* variants) had CADD scores > 20, indicating a higher probability of a deleterious effect (**Tables 1**–**3**). In addition, looking at variant burden in population, we found that 98% (54/55) of the identified RPA heterotrimer variants had AF < 0.1% (this includes rare, very-rare, ultra-rare, and novel variants, **Figure 1B**). All *RPA1*, *RPA2* and *RPA3* variants are mutually exclusive and no cases with compound heterozygous or homozygous variants were identified.

**Table 1:** Germline heterozygous variants found in RPA1, RPA2 and RPA3 in pediatric hematological malignancies.

Genomic position	SJID	Diagnosis	Age	RPA1 Domain	Heterozygous RPA1 germline variant	Genetic Ancestry	gnomad non-cancer v.2.1.1 AF	Ancestry specific AF	CADD	REVEL	InterVar automated classification	Other heterozygous germline variants	Somatic mutations	Stability (ccal/mol)
1747265	SJBALL032225	BALL	9.18	F	c.A136G:p.M46V	AMR	0.0013%	0.0029%	22.2	0.28	VUS	none reported	IKZF1 del	unavailable
1747266	SJALL041240	BALL	4.13	F	c.T137C:p.M46T	NFE	novel	novel	25.2	0.456	VUS	none reported	NA	1.9
1747283	SJALL041360	BALL	14.08	F	c.155_156del:p.L53Ifs*53	NFE	0.0008%	0.0020%	NA	NA	VUS	none reported	NA	unavailable
1747879	SJHL042034	HL	17.4	F	c.G171A:p.M57I	NFE	novel	novel	28	0.492	VUS	none reported	NA	-0.2
1747882	SJCBF147	AML	17.68	F	c.G174C:p.L58F	NFE	0.0009%	0.0010%	25.2	0.489	VUS	none reported	NRAS	1.2
1780546	SJTALL021675	TALL	22.38	A	c.C628T:p.R210C	Other	0.0038%	novel	35	0.594	VUS	none reported	none reported	0.6
1782352	SJBALL020994	BALL	24.38	A	c.C756G:p.N252K	Other	0.0063%	0.0178%	26.6	0.267	VUS	none reported	RCSD2-ABL2 fusion, IKZF1 deletion, VPREB deletion	0.2
1782605	SJAML030416	AML	17.11	A	c.G856T:p.V286F	Other	0.0097%	0.0149%	33	0.373	VUS	NOTCH2 (p.P6fs*, novel); FANCD2 (p.V427_E15splice, novel)	TP53(p.D281H), ETV6(p.F417fs), WT1(p.R414fs), PHF6(p.R225*), FLT3(p.Y597>11aa)	0.6
1782605	SJAML031075	AML (AMKL)	3.13	A	c.G856A:p.V286I	AMR	0.0410%	novel	16.3	0.373	VUS	MLL (p.I882fs)	JAK1:p.L783F;JAK3:p.A573V;GATA1:p.S30_G31fs;STAG2:p.T149fs	0.6
1782605	SJAML032052	AML (AMML)	16.23	A	c.G856A:p.V286I	NFE	0.0410%	0.0286%	16.3	0.373	VUS	none reported	NPM1:p.W288fs; PTPN11:p.E76K	0.6
1782605	SJAML032355	AML (AMML)	17	A	c.G856A:p.V286I	NFE	0.0410%	0.0286%	16.3	0.373	VUS	HIP1:Amplification	ERCC2:p.M1fs;NPM1:p.W288fs;NRAS:p.G12D;PTPN11:p.A72V;TRIM28:p.I302_K304fs;SLC45A3_ELK4:Fusion	0.6
1782605	SJALL016427	BALL	NA	A	c.G856T:p.V286F	NFE	0.0097%	0.0203%	33	0.373	VUS	none reported	none reported	0.6
1782983	SJALL041859	BALL	13.99	B	c.G1082T:p.W361L	NFE	0.0008%	0.0019%	32	0.752	VUS	none reported	NA	2.2
1783867	SJHL041557	HL	NA	B	c.G1123A:p.V375M	NFE	0.0021%	0.0049%	28.6	0.413	VUS	none reported	NA	1.2
1783909	SJTALL022093	TALL	3.02	B	c.C1165T:p.R389W	AMR	0.1763%	0.0028%	35	0.319	VUS	none reported	none reported	-0.9
1783909	SJBALL001702	BALL	NA	B	c.C1165T:p.R389W	NFE	0.1763%	0.0736%	35	0.319	VUS	none reported	none reported	-0.9
1783909	SJALL015269	TALL	6.3	B	c.C1165T:p.R389W	NFE	0.1763%	0.0736%	35	0.319	VUS	none reported	none reported	-0.9
1783909	SJBALL032592	BALL	12.78	B	c.C1165T:p.R389W	NFE	0.1763%	0.0736%	35	0.319	VUS	ATM (p.W2769*, 0.0008%)	TACC3-FGFR3: focal amplification	-0.9
1787123	SJNHL042753	NHL	7.24	B	c.A1259T:p.Q420L	NFE	0.0019%	0.0042%	22	0.16	VUS	none reported	NA	unavailable
1787161	SJNHL042070	NHL	14.35	coding, non DBD	c.G1297A:p.G433S	AFR	0.0157%	0.0508%	23.5	0.218	VUS	none reported	NA	unavailable
1787173	SJHL042469	HL	11.31	C	c.G1309A:p.G437R	AFR	0.0134%	0.1397%	24.4	0.286	VUS	none reported	NA	unavailable
1792053	SJALL018944	BALL	19.48	C	c.A1459G:p.N487D	NFE	novel	novel	28.6	0.564	VUS	none reported	NA	0.2
1792111	SJPHALL020033	BALL	3.28	C	c.A1517T:p.E506V	AMR	0.0034%	novel	32	0.387	VUS	BRIP1 (p.P47A, 0.03%), TAL1 (p.E1_splice, novel)	none reported	-0.6
1792111	SJALL015640	TALL	5.23	C	c.A1517T:p.E506V	AFR	0.0034%	0.0381%	32	0.387	VUS	none reported	none reported	-0.6
1792132	SJERG020054	BALL	NA	C	c.G1538A:p.R513H	NFE	0.0164%	0.0331%	35	0.535	VUS	none reported	IKZF1 del, CDKN2A del, ETV6 del	0.4
1792132	SJHL019322	HL	15.53	C	c.G1538A:p.R513H	NFE	0.0164%	0.0331%	35	0.535	VUS	none reported	NA	0.4
1795196	SJALL041325	BALL	11.21	C	c.G1621A:p.G541R	NFE	novel	novel	26.1	0.591	VUS	none reported	NA	0.1
1795196	SJNHL018781	NHL	14.7	C	c.G1621A:p.G541R	NFE	novel	novel	26.1	0.591	VUS	CTC1 (p.R224*, 0.00008%)	NA	0.1
1800399	SJALL018992	BALL	7.78	C	c.T1781G:p.V594G	NFE	0.0011%	0.0013%	26.5	0.569	VUS	RAD51D(p.G258fs*,novel)	NA	3.3

Genomic position	SJID	Diagnosis	Age	RPA2 Domain	Heterozygous RPA2 germline variant	Genetic Ancestry	gnomad non-cancer v.2.1.1 AF	Ancestry specific AF	CADD	REVEL	InterVar automated classification	Other heterozygous germline variants	Somatic mutations	Stability (ccal/mol)
28233784	SJHYPO109	BALL	2.11	coding, non DBD	c.G127A:p.A43T	NFE	0.0032%	0.0065%	17.1	0.039	VUS	none reported	none reported	unavailable
28233735	SJTALL022654	TALL	11.66	D	c.T176G:p.L59W	Other	0.0022%	0.0299%	22.6	0.131	VUS	TSC1(p.E876_E21splice, novel)	none reported	0.1
28233489	SJHL041547	HL	14.62	D	c.G283A:p.D95N	NFE	0.0004%	0.0010%	34	0.409	VUS	POLG (p.R1096C, 0.001%)	NA	-0.2
28223548	SJHL041567	HL	16.45	D	c.G493A:p.A165T	NFE	novel	novel	27.8	0.108	VUS	none reported	NA	0.2

Genomic position	SJID	Diagnosis	Age	RPA3 Domain	Heterozygous RPA3 germline variant	Genetic Ancestry	gnomad non-cancer v.2.1.1 AF	Ancestry specific AF	CADD	REVEL	InterVar automated classification	Other heterozygous germline variants	Somatic mutations	Stability (ccal/mol)
7680021	SJAML042701	AML	0.65	E	c.C29T:p.S10L	NFE	novel	novel	23.2	0.025	VUS	RTEL1 (p.R986*, novel)	NA	-1.4
7679976	SJALL003857	BALL	1.84	E	c.T74C:p.V25A	Other	novel	novel	29.1	0.506	VUS	SH3B2 (p.G470_E8splice, novel)	NA	3.8
7679965	SJALL041268	BALL	4.05	E	c.G85C:p.G29R	NFE	0.0032%	0.0065%	34	0.775	VUS	none reported	NA	0.5
7678756	SJHL041577	HL	11.83	E	c.118delA:p.M40Cfs*16	NFE	novel	novel	NA	NA	VUS	none reported	NA	unavailable
7678736	SJTALL022645	TALL	NA	E	c.G139A:p.E47K	NFE	0.0008%	0.0020%	26.6	0.222	VUS	none reported	none reported	0.3
7677512	SJALL041310	BALL	1.91	E	c.A266G:p.E89G	EAS	0.0004%	0.0057%	27.3	0.33	VUS	none reported	NA	1.9

BALL, B-cell acute lymphoid leukemia; TALL, T-cell acute lymphoid leukemia ALL; AML, acute myeloid leukemia; HL, Hodgkin’s lymphoma; NHL, non-Hodgkin’s lymphoma; AMR, Admixed/Latino; NFE, Non-Finnish European; AFR, African; EAS, East Asian; VUS, variant of unknown significance; NA, not available; unavailable, lack of structural coverage or accuracy at nucleotide position.

**Table 2:** Germline heterozygous variants found in RPA1, RPA2 and RPA3 in extra-cranial solid tumors.

Genomic position	SJID	Diagnosis	Age	RPA1 Domain	Heterozygous RPA1 germline variant	Genetic Ancestry	gnomad non-cancer v.2.1.1 AF	Ancestry specific AF	CADD	REVEL	InterVar automated classification	Other heterozygous germline variants	Somatic mutations	Stability (ccal/mol)
1747265	SJNBL017162	NBL	4.69	F	c.A136G:p.M46V	NFE	0.0013%	0.0010%	22.2	0.28	VUS	none reported	none reported	0.1
1756468	SJRHB032408	Sarcoma (RMS)	3.1	coding, non DBD	c.G346A:p.V116M	AMR	novel	novel	23.4	0.151	VUS	CNOT3 (p.P243fs, novel)	BCOR(p.F1385fs)	0.6
1779063	SJWLM019906	WT	4.9	A	c.C563T:p.A188V	NFE	0.0161%	0.0078%	23.7	0.136	VUS	none reported	none reported	-0.4
1780603	SJNBL030203	NBL	4.44	A	c.G685A:p.E229K	AFR	0.0004%	novel	31	0.227	VUS	TP53 (p.A161T, novel)	ALK(p.R1275Q)	0
1782605	SJSTS019601	MEL	9.9	A	c.G856A:p.V286I	NFE	0.0403%	0.0254%	16.32	0.206	VUS	BRIP1 (p.Q685*, 0.006%), MED12 (p.Q2109_Q2115>Q, 0.0009%)	none reported	unavailable
1782605	SJMEL031366	Sarcoma (RMS)	11.42	A	c.G856A:p.V286I	NFE	0.0403%	0.0254%	16.32	0.206	VUS	NA	NA	0.1
1782605	SJRHB000026	Sarcoma (synovial)	9.9	A	c.G856A:p.V286I	NFE	0.0403%	0.0254%	16.32	0.206	VUS	none reported	NA	-0.2
1782611	SJGCT019774	GCT	16.34	A	c.C862T:p.P288S	AFR	novel	novel	31	0.367	VUS	none reported	NA	0.4
1783909	SJRB030058	RB	0.28	B	c.C1165T:p.R389W	NFE	0.1763%	0.0736%	35	0.319	VUS	none reported	none reported	-0.6
1783972	SJOS040162	Sarcoma (OS)	22.78	B	c.A1228G:p.K410E	Other	novel	novel	23.3	0.148	VUS	none reported	none reported	0.1
1787123	SJST032198	PTC	18.13	B	c.A1259T:p.Q420L	NFE	0.0019%	0.0042%	22	0.16	VUS	NTHL1 (p.A237_E5splice, novel)	BRAF(p.V600E)	0.8
1787140	SJEWS019204	Sarcoma (EWS)	16.11	coding, non DBD	c.T1276G:p.S426A	NFE	0.0021%	0.0049%	24.1	0.191	VUS	none reported	NA	-0.9
1787161	SJNBL017202	NBL	1.51	coding, non DBD	c.G1297A:p.G433S	AFR	0.0157%	0.0508%	23.5	0.218	VUS	PALB2 (p.G562_E4splice, novel), NDRG4 (p.M292_E14splice, novel)	none reported	0
1787173	SJSTS042513	Sarcoma (synovial)	8.53	C	c.G1309A:p.G437R	AFR	0.0134%	0.1397%	24.4	0.286	VUS	none reported	NA	unavailable
1787173	SJSTS019626	WT	0.87	C	c.G1309A:p.G437R	AFR	0.0134%	0.1397%	24.4	0.286	VUS	none reported	NA	0.4
1792008	SJRB019561	RB	2.34	C	c.C1414T:p.R472C	AFR	0.0026%	0.0212%	35	0.542	VUS	RB1 (p.L218*,novel)	NA	-0.2
1792111	SJNPC019502	NPC	13.81	C	c.A1517T:p.E506V	AFR	0.0034%	0.0381%	32	0.387	VUS	none reported	NA	0.6
1792120	SJRB017939	RB	0.17	C	c.A1526G:p.N509S	NFE	0.0008%	novel	20.6	0.141	VUS	RB1 (p.R358*, novel)	NA	0.2
1795196	SJNBL018730	NBL	0.11	C	c.G1621A:p.G541R	NFE	novel	novel	26.1	0.591	VUS	MDC1 (p.A710_E6splice, novel)	NA	0.6
1795226	SJNBL017207	NBL	2.42	C	c.A1651C:p.K551Q	NFE	novel	novel	22.7	0.282	VUS	none reported	none reported	unavailable
1800386	SJOS018814	Sarcoma (OS)	11.76	C	c.A1768G:p.T590A	AMR	0.0050%	0.0325%	23.1	0.15	VUS	none reported	NA	-0.2
1800407	SJNBL042729	NBL	0.93	C	c.G1789A:p.V597M	NFE	0.0011%	0.0014%	27.8	0.292	VUS	none reported	NA	unavailable

Genomic position	SJID	Diagnosis	Age	RPA2 Domain	Heterozygous RPA2 germline variant	Genetic Ancestry	gnomad non-cancer v.2.1.1 AF	Ancestry specific AF	CADD	REVEL	InterVar automated classification	Other heterozygous germline variants	Somatic mutations	Stability (ccal/mol)
28233735	SJOS040159	Sarcoma (OS)	19.92	D	c.T176G:p.L59W	Other	0.0022%	0.0299%	22.6	0.131	VUS	none reported	none reported	0.1
28233504	SJRB041658	RB	0.24	D	c.A268G:p.I90V	NFE	0.0004%	0.0010%	18.28	0.077	VUS	RB1 (p.E237*, novel)	NA	unavailable
28240645	SJNBL017483	NBL	3.88	coding, non DBD	c.G46A:p.G16R	AFR	0.0008%	0.0136%	22.6	0.088	VUS	none reported	none reported	unavailable

Genomic position	SJID	Diagnosis	Age	RPA3 Domain	Heterozygous RPA3 germline variant	Genetic Ancestry	gnomad non-cancer v.2.1.1 AF	Ancestry specific AF	CADD	REVEL	InterVar automated classification	Other heterozygous germline variants	Somatic mutations	Stability (ccal/mol)
7679965	SJLPS014753	Sarcoma (lipo)	22	E	c.G85C:p.G29R	NFE	0.0032%	0.0065%	34	0.775	VUS	NA	NA	0.5
7676696	SJWLM018894	WT	1.05	E	c.G301A:p.E101K	AFR	0.0015%	0.0085%	34	0.325	VUS	WT1 (p.Q238*, novel)	NA	0
7676669	SJWLM043921	WT	1.05	E	c.C328G:p.P110A	NFE	0.0064%	0.0130%	23.1	0.512	VUS	WT1:CNV Del, LIG4 (p.K424fs, novel)	NA	0.3

GCT, germ cell tumor; MEL, melanoma; NBL, neuroblastoma; NPC, nasopharyngeal carcinoma; PTC, papillary thyroid carcinoma; EWS, Ewing’s sarcoma; OS, osteosarcoma; RMS, rhabdomyosarcoma; WT, Wilms tumor; AMR, Admixed/Latino; NFE, Non-Finnish European; AFR, African; VUS, variant of unknown significance; NA, not available; unavailable, lack of structural coverage or accuracy at nucleotide position.

**Table 3:** Germline heterozygous variants found in RPA1, RPA2 and RPA3 in extra-cranial solid tumors.

Genomic position	SJID	Diagnosis	Age	RPA1 Domain	Heterozygous RPA1 germline variant	Genetic Ancestry	gnomad non-cancer v.2.1.1 AF	Ancestry specific AF	CADD	REVEL	InterVar automated classification	Other heterozygous germline variants	Somatic mutations	Stability (ccal/mol)
1778987	SJLGG031132	LGG (Ganglioglioma)	11.23	coding, non DBD	c.A487G:p.K163E	NFE	0.0008%	0.0010%	16.67	0.128	VUS	none reported	BRAF:p.V600E	unavailable
1782296	SJEPD030782	EPD	3.98	A	c.C700G:p.R234G	NFE	0.0008%	0.0019%	28	0.598	VUS	none reported	none reported	1.5
1782605	SJMB030776	MB	3.86	A	c.G856T:p.V286F	AMR	0.0097%	novel	33	0.373	VUS	PBRM1 (p.K128_E4splice, novel)	none reported	-0.4
1782646	SJMB032506	MB	12.8	coding, non DBD	c.G897C:p.Q299H	NFE	novel	novel	22.4	0.081	VUS	C7 (p.R521S, 0.002%); MYH9 (p.F235_E6splice, novel)	none reported	-0.2
1787173	SJHGG117	HGG	2.57	C	c.G1309A:p.G437R	AFR	0.0134%	0.1397%	24.4	0.286	VUS	none reported	none reported	unavailable
1787173	SJHGG030703	HGG (HGNET)	1.49	C	c.G1309A:p.G437R	AFR	0.0134%	0.1397%	24.4	0.286	VUS	none reported	NUTM2B_Deletion	unavailable
1792075	SJHGG067	HGG	5.6	C	:c.A1481G:p.Q494R	EAS	novel	novel	23.5	0.202	VUS	none reported	none reported	-0.1
1792089	SJLGG030365	LGG	1.11	C	c.C1495T:p.R499C	AMR	0.0052%	0.0171%	35	0.435	VUS	SDHA (p.R31*, 0.02%) RUNX1 (p.Q415*, 0.0006%)	KIAA1549_BRAF_Fusion	-0.1
1792113	SJCNS018575	MB	9.19	C	c.T1519C:p.F507L	NFE	novel	novel	28.7	0.179	VUS	NA	NA	0.6
1792132	SJLGG046	LGG	5.1	C	c.G1538A:p.R513H	NFE	0.0164%	0.0331%	35	0.535	VUS	none reported	none reported	0.4
1792139	SJHGG100	HGG	10.99	C	c.C1545G:p.I515M	NFE	0.0008%	novel	17.81	0.074	VUS	none reported	none reported	0.5
1800398	SJST032495	MB	14	C	c.G1780A:p.V594M	NFE	novel	novel	33	0.419	VUS	BRCA1 (p.E1559_E15splice, novel)	NA	0.7

Genomic position	SJID	Diagnosis	Age	RPA3 Domain	Heterozygous RPA1 germline variant	Genetic Ancestry	gnomad non-cancer v.2.1.1 AF	Ancestry specific AF	CADD	REVEL	InterVar automated classification	Other heterozygous germline variants	Somatic mutations	Stability (ccal/mol)
7676696	SJMB031439	MB	9.37	E	c.G301A:p.E101K	EAS	0.0015%	0.0104%	34	0.325	VUS	ANKRD26 (p.Y1708*), novel	PTCH1(p.Y93fs), AFF4 truncating insertion; CNVs- PTEN, SMARCA2, JAK2	0

EP, ependymoma; LGG, low grade glioma; MB, medulloblastoma; HGG, high grade glioma; AMR, Admixed/Latino; NFE, Non-Finnish European; AFR, African; EAS, East Asian; VUS, variant of unknown significance; NA, not available; unavailable, lack of structural coverage or accuracy at nucleotide position.

### *RPA1* germline variants and cancers

RPA1 (616 amino acids, 70kDa) is the largest of the 3 subunits of the RPA heterotrimer. We discovered 1.05% (63/5993) of the cohort to harbor heterozygous germline *RPA1* variants (**Figure 1C**; **Tables 1**–**3**), which was statistically not significant compared to gnomAD non-cancer controls for all cancers and cancer subtypes (**Table 4**). RPA1 has 4 modular oligosaccharide binding-fold domains commonly referred to as functional DNA binding domains (DBD): F, A, B and C spanning the N- to C- terminal regions of the protein. *RPA1* variants were found across all 4 DBDs as follows: 6 in DBD-F, 15 in DBD-A, 10 in DBD-B, and 26 in DBD-C (**Figure 1C**). Of note, 6 cases were found to have *RPA1* variants in the linker regions between 2 DBDs. All *RPA1* variants were missense (**Figure 1C**) except for p.L53lfs*53 within DBD-F, which was found in 1 case. Three recurrently mutated amino acids were discovered in RPA1 domains DBD-A (p.V286, 9 cases), DBD-B (p.R389, 5 cases), and DBD-C (p.G437, 5 cases). We next focused specifically on novel and ultra-rare *RPA1* variants (33), present in 14 and 21 cases, respectively (**Figure 1B**; **Tables 1**–**3**). Notably, we found significant enrichment of *RPA1* novel and ultra-rare variants in our cohort (adjusted p-value < 0.05, **Table 4**).

Prediction of variant structural effect was performed by calculating protein stability change scores (ΔΔG_Fold_) with a ΔΔG_Fold_ that affects the ratio of folded to unfolded protein 12-fold (~1.5 kcal/mol) for both stabilizing and destabilizing mutations. Significant scores (>1.5 kcal/mol) were demonstrated for 4 variants (p.M46T in DBD-F, p. R234G in DBD-A, p.W361L in DBD-B, p.V594G in DBD-C) which were novel or ultra-rare (**Tables 1**–**3**). RPA1 p.M46T is likely to destabilize folding of DBD-F resulting in the loss of multiple important protein-protein interactions (11). W361 is a key DNA binding residue in DBD-B and human cells with W361A support normal replication but are deficient in DNA repair (12, 34), suggesting that p.W361L may destabilize DBD-B folding resulting in hypomorphic RPA.

We next assessed which types of malignancies were present in patients with *RPA1* variants (**Figure 1A**). We found comparable frequency of cases with *RPA1* variants across solid tumors (n = 22, 1.1%), CNS cancers (n = 12, 1.1%) and hematological malignancies (n = 29, 0.8%). Among solid tumor cases with *RPA1* variants, 31.8% (7/22) presented with sarcomas and 27.3% (6/22) were diagnosed with neuroblastoma. Notably, the 7 sarcoma cases carried 6 unique *RPA1* variants (n = 2 novel and n = 1 ultra-rare) and only one was noted to have a concomitant germline mutation (**Table 2**). All 6 neuroblastoma cases were found to have unique *RPA1* variants (n = 3 novel, n = 2 ultra-rare) of which half were found to have germline variants reported in *PALB2*/*NDRG4*, *MDC1*, or *TP53* genes (**Table 2**). Three cases of retinoblastoma harbored unique *RPA1* variants (2 ultra-rare) with 2 cases having concomitant germline *RB1* mutation (**Table 2**). Two cases of Wilms tumor were identified to have germline *RPA1* variants. Among the single cases of solid tumors (germ cell tumor, melanoma, nasopharyngeal carcinoma, and papillary thyroid carcinoma), 2 ultra-rare and 1 novel *RPA1* variants were found.

Among cases with CNS tumors, 4 patients with medulloblastoma harbored novel (n = 3) or ultra-rare (n = 1) *RPA1* variants. Each of these cases also carried other germline mutations (*PBRM1*, *C7* and *MYH9*, *BRCA1*, *ANKRD26*) of which *BRCA1* and *ANKRD26* are cancer predisposition genes (**Table 3**). Furthermore, 4 cases with high grade glioma harbored 3 *RPA1* variants (n = 1 novel, n = 1 ultra-rare), all clustering within DBD-C domain of RPA1. These patients had no other potentially causative germline variants reported in other predisposition genes. Among the 3 low grade glioma, 3 *RPA1* variants (one ultra-rare) were identified, with one harboring other germline mutations in *SDHA* and *RUNX1*. Lastly, one ultra-rare *RPA1* variant was identified in a case of ependymoma without other germline mutations (**Table 3**).

From patients with hematologic malignancies, *RPA1* variants were most common in B-ALL (n = 13), followed by lymphoma (n = 7), AML (n = 5), and T-ALL (n = 4) (**Table 1**). Out of 13 B-ALL cases, 3 and 5 were novel and ultra-rare, respectively. Only 2 cases out of the 13 had heterozygous germline variants in cancer predisposition genes (*RAD51D*, *BRIP1)*. Among lymphomas, we observed 4 Hodgkin’s lymphoma (n = 1 novel, n = 1 ultra-rare) and 3 non-Hodgkin’s lymphoma (n = 1 novel, n = 1 ultra-rare) with *RPA1* variants. In the AML sub-cohort, we found 3 unique *RPA1* variants in 5 cases, of which 4 were mutated at nucleotide 856 in DBD-A (c.856G>T, c.856G>A coding different amino acids) and 1 ultra-rare variant in DBD-F domain (**Table 1**). Of the 5 *RPA1*-mutated AML cases, 3 carried other germline variants (MLL, HIP1, NOTCH2 and FANCD2). Four unique *RPA1* variants (n = 2 ultra-rare) were discovered in 4 patients with T-ALL, with one case having additional germline *ATM* variant (**Table 1**).

Given the occurrence of MDS/AML in one prior patient with germline *RPA1* p.V227A with TBD (10) and 5 AML cases in this study, we queried a cohort of 41 young adults with AML and karyotype aberrations (18) for RPA heterotrimer germline variants. We found 1 ultra-rare (c.460G>A, T154A, AF 0.001%) and 2 rare (c.1397C>G, A466G, AF 0.027%; c.1538G>A, R513H, AF 0.016%) *RPA1* heterozygous variants (**Supplemental Table 1**).

### *RPA2 and RPA3* germline variants and cancers

RPA2 is the second largest subunit (270 amino acids, 34kDa) of the RPA heterotrimer. We identified 6 heterozygous germline *RPA2* variants in 7 cases of pediatric malignancies. Five variants are present in DBD-D (**Figure 1D**) and did not exhibit dysfunctional protein folding scores (**Tables 1**, **2**). All variants were either ultra-rare (n = 5) or novel (n = 1). Four patients (4/3452, 0.1%) had hematological malignancies (n = 1 B-ALL, n = 1 T-ALL, n = 2 Hodgkin’s lymphoma) and 3 had solid cancers (n = 1 RBL, n = 1 neuroblastoma, n = 1 sarcoma). Other germline mutations were noted in 3 out of 7 cases (**Tables 1**, **2**).

RPA3, although less than half the size of RPA2 (121 amino acids, 14kDa) had 8 unique germline heterozygous variants (n = 4 ultra-rare, n = 3 novel) in 10 cases of pediatric cancers, including 6 hematologic (B-ALL n = 3, T-ALL n = 1, AML n = 1, Hodgkin’s lymphoma n = 1), 3 solid tumors (Wilms tumor n = 2, sarcoma n = 1) and 1 CNS (medulloblastoma) cancers (**Figure 1E**; **Tables 1**–**3**). All were missense except for one frameshift (p.M40Cfs*16). Protein folding scores for 2 out of 7 available *RPA3* variants were greater than 1.5 kcal/mol and were either novel or ultra-rare (**Tables 1**–**3**). Half of *RPA3* mutated cases had other germline variants noted (**Tables 1**–**3**). The number of cases with *RPA2* or *RPA3* germline variants did not reach statistical significance compared to gnomAD non-cancer controls (**Table 4**).

**Table 4:** Statistical analysis using two- and one-sided Fisher exact tests of ultra-rare plus novel and rare germline heterozygous variants in RPA1, RPA2 and RPA3 across hematologic, extra-cranial solid and CNS tumors.

Ultra-rare or novel variants AF<0.005%												
Gene	Subset	Cancer_AF	Control_AF	Cancer_Alt_Count	Cancer_Total_Count	Control_Alt_Count	Control_Total_Count	p.value (fisher.test-greater)	OR (fisher.test-greater)	FDR_fisher_greater	p.value (Two-sided)	FDR_fisher_twosided
RPA1	PanCancer_UltraRare	0.0029	0.0017	35	11951	466	267908	0.00350858	1.6837	0.028068639	0.00531368	0.042509437
RPA1	HEM_UltraRare	0.0022	0.0017	15	6889	466	267908	0.231331347	1.2518	0.462662694	0.37871645	0.504955267
RPA1	ST_UltraRare	0.0033	0.0017	13	3935	466	267908	0.024524772	1.8993	0.09809909	0.031863803	0.127455213
RPA1	CNS_UltraRare	0.0033	0.0017	7	2129	466	267908	0.083930393	1.8903	0.223814382	0.107377392	0.214754784
RPA2	PanCancer_UltraRare	0.0006	0.0007	7	11979	200	268174	0.785228312	1	0.729135903	0.7835	0.927224074
RPA3	PanCancer_UltraRare	0.0008	0.0006	9	11977	164	268210	0.32195265	1	0.568945016	1.2289	0.853417524

All variants AF<0.5%												
Gene	Subset	Cancer_AF	Control_AF	Cancer_Alt_Count	Cancer_Total_Count	Control_Alt_Count	Control_Total_Count	p.value (fisher.test-greater)	OR (fisher.test-greater)	FDR_fisher_greater	p.value (Two-sided)	FDR_fisher_twosided
RPA1	PanCancer	0.0053	0.0061	63	11923	1614	266760	0.868322713	0.8733	0.982140829	0.33224587	0.504955267
RPA1	HEM	0.0042	0.0061	29	6875	1614	266760	0.982140829	0.6972	0.982140829	0.056909619	0.151758983
RPA1	ST	0.0056	0.0061	22	3926	1614	266760	0.667688785	0.9262	0.890251714	0.835352152	0.954688173
RPA1	CNS	0.0056	0.0061	12	2124	1614	266760	0.631653729	0.9338	0.890251714	1	1
RPA2	PanCancer	0.0006	0.0078	7	11979	2081	266293	1	1	4.73434E-30	0.0748	7.10151E-29
RPA2	HEM	0.0006	0.0078	4	6900	2081	266293	1	1	5.16066E-18	0.0742	3.87049E-17
RPA2	ST	0.0008	0.0078	3	3945	2081	266293	1	1	5.40401E-10	0.0973	2.70201E-09
RPA2	CNS	0	0.0078	0	2136	2081	266293	1	1	1.34364E-07	0	5.03865E-07
RPA3	PanCancer	0.0008	0.0006	10	11976	169	268205	0.237746122	1	0.353635923	1.3252	0.663067356
RPA3	HEM	0.0009	0.0006	6	6898	169	268205	0.276876834	1	0.459624798	1.3804	0.76604133
RPA3	ST	0.0008	0.0006	3	3945	169	268205	0.455502374	1	0.741779259	1.2068	0.927224074
RPA3	CNS	0.0005	0.0006	1	2135	169	268205	0.740267746	1	1	0.7433	1

“PanCancer”, all cancers in the cohort; HEM, hematologic; ST, solid tumor; CNS, central nervous system.

## Discussion

The RPA heterotrimer is an essential protein for binding ssDNA encountered in cellular transactions to facilitate DNA-DNA and DNA-protein interactions during DNA replication, repair, recombination, RNA transcription, and telomere maintenance. As such, mutations in this genome maintenance protein have been linked to cancer formation in mice (35) and are acquired in up to ~1% of human cancers (14). We recently demonstrated that heterozygous germline *RPA1* mutations *RPA1* c.680T>C p.V227A, c.718G>A p.E240K and c.808A>G p.T270A in DBD-A are associated with TBD, which predisposes to hematologic and solid tumors. In this study, one patient with RPA1-related TBD developed MDS (10). Based on these data, we reasoned that germline defects in RPA1 and possibly also the other 2 components of the RPA heterotrimer (RPA2 and RPA3) might be associated with cancer development. To this end, we investigated comprehensive germline genomic data for the presence of heterozygous variants in *RPA1*, *RPA2* and *RPA3* across a large series of pediatric hematologic, solid and CNS malignancies. We discovered significant enrichment of ultra-rare and novel *RPA1* germline variants in our pediatric cancer cohort compared to non-cancer controls, positioning *RPA1* as a novel candidate predisposition gene. Moreover, in an additional cohort of 41 patients with AML, we identified 3 heterozygous germline *RPA1* variants (c460G>A, p.T154A; c.1397C>G, p.A466G; c.1538G>A, p.R513H) with potential pathogenic effect.

*RPA1* harbored the most variants likely due to its larger size compared to *RPA2* and *RPA3*. Although we did not observe a statistically significant enrichment of putative damaging variants in *RPA2* and *RPA3*, some of the identified variants were novel or ultra-rare and could possibly have a deleterious effect. Thus, *RPA2* and *RPA3* could be considered as genes of unknown significance (GUS) yet potentially important in tumor formation. All 3 proteins are required to fold properly to form a functional RPA heterotrimer (13). For this reason, we calculated stabilities of the RPA modular domains harboring mutations to gain insight into the possible effect of identified germline variants on RPA heterotrimer function. Scores greater than 1.5 are highly predictive of protein instability and dysfunction. High protein folding scores were found for 4 unique *RPA1* variants in 4 cases, 3 identified in patients with B-ALL and one in a patient with ependymoma. All were either ultra-rare or novel with CADD scores suggesting high likelihood of pathogenicity. Two *RPA3* variants also harbored high protein folding scores in patients with B-ALL. This suggests that dysfunctional folding of the RPA heterotrimer may lead to genomic instability in these patients.

In our discovery cohort, we identified 5 AML cases with germline *RPA1* variants. One had an ultra-rare *RPA1* p.L58F variant in DBD-F and the remaining 4 had variants affecting nucleotide 856 within DBD-A domain (c.G856A, p.V286I in 3 cases and c.G856T, p.V286F in one case). The resulting amino acid changes do not differ in size or charge from wild-type valine and have a neutral protein folding score of 0.6. However, these mutations may disrupt protein-protein, protein-DNA interactions, or post-translational modifications, which are known mechanisms implicated in pathogenicity of *RPA1* variants in various experimental models (10–13, 35). Additionally, TBD-associated pathogenic *RPA1* variants, p.V227A, p.E240K and p.T270A, have protein folding scores of 1.4, 0.1 and 0.2 (consistent with normal protein folding shown in biochemical assays) yet were shown to exert gain-of-function effect on DNA binding and melting of telomeric G-quadruplexes (10). Three of the 4 AML cases with *RPA1* variants in DBD-A domain had additional germline variants in genes (NOTCH2, FANCD2, MLL, HIP1) which, together with *RPA1* may have an epistatic effect to cause overall genomic instability. Corroborating data from a small cohort of 41 AML patients in which 3 patients carried *RPA1* variants (p.T154A in linker region; p.A466G and p.R513H in DBD-C) deserves further investigation. Beyond *RPA1* in the AML cohort, we also found a novel germline missense variant in *RPA3* in an infant with AML who also harbored a germline truncating variant in the DNA helicase, *RTEL1*, which is associated with TBD (36, 37). More functional studies are needed to determine the pathogenicity of *RPA1* V286I/F alterations and their role in hematologic malignancy.

Among the 13 CNS tumors with variants in RPA heterotrimer genes, 9 cases were high grade neoplasms, including medulloblastoma and high-grade glioma. Interestingly, 3 of the 5 medulloblastoma cases had novel and one very rare germline *RPA1*, as well as one ultra-rare *RPA3* variant. Notably, even though variants in other unrelated genes were also found in 4 of the 5 medulloblastoma cases, none of these genes have been previously associated with medulloblastomas in the literature. Other studies have identified germline defects in DNA repair genes in medulloblastoma (38, 39). It would stand to reason that germline mutations in the RPA heterotrimer, which functions in almost all DNA repair pathways, could potentiate oncogenic transformation. Further investigation should focus on assessing the function of RPA mutant proteins in DNA repair and their contribution to tumor biology.

Our study has several limitations. Although all cases were assessed using a uniform pipeline, the cohort is skewed towards cases with B-ALL (~4-fold higher number of B-ALL compared to solid and CNS cancers). We included all germline and somatic mutations per case that were reported in previously published studies; however, this information was unavailable for a proportion of cases and therefore we cannot make definitive conclusions about *RPA* variants being the sole germline driver in these cancers. Although ultra-rare and novel heterozygous germline variants in *RPA1* were significantly enriched in pediatric cancers, it is difficult to ascertain pathogenicity and clinical relevance without functional follow-up, which falls beyond the scope of this study. It is plausible that variants with high in-silico protein folding energy, ultra-rare and/or novel allelic frequency and high pathogenicity scores may be clinically relevant and should be among the top variants to explore in future studies.

In summary, evasion of DNA repair mechanisms is a common theme among cancers. RPA is an essential protein for DNA replication and repair. Our study describes novel and rare variants with potentially deleterious effect in the *RPA1*, *RPA2* and *RPA3* genes in pediatric malignancies. Moreover, we have identified enrichment of *RPA1* variants in cancer cases compared to non-cancer controls, suggesting that this gene potentially acts as a novel cancer driver. We plan to exploit our findings and perform further functional and biochemical characterization of recurrent cancer associated *RPA1* variants to assess their potential use as targets for future cancer therapies.

## Data availability statement

The datasets presented in this study can be found in online repositories. The names of the repository/repositories and accession number(s) can be found in the article/**Supplementary Material**.

## Author contributions

RS, KN, and MW: conceptual design of the study and data interpretation. NO and WC: data analysis and statistics. RG, MJS, and MS: computational analysis of RPA mutations and interpretation. MK and FB: conceptual design and interpretation. All authors contributed to manuscript preparation and editing. All authors approved the submitted version.
