# Supplementary material for: Germline landscape of RPA1, RPA2 and RPA3 variants in pediatric malignancies: identification of RPA1 as a novel cancer predisposition candidate gene
Source: Front Oncol. 2023 Oct 6;13:1229507. doi: 10.3389/fonc.2023.1229507 (PMC10588448; doi:10.3389/fonc.2023.1229507)
Supplement: Supplementary file 1 [file Table_1.docx]

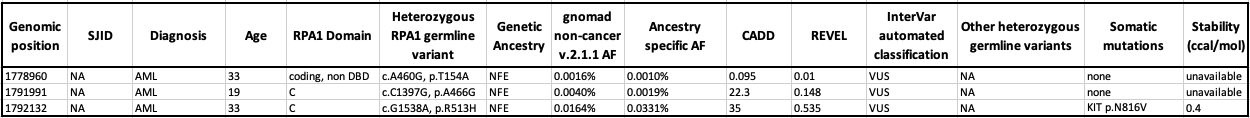


**Supplemental Table 1- Germline *RPA1* heterozygous variants found in a cohort of 41 young adult patients with AML.** AML, acute myeloid leukemia; NFE, Non-Finnish European; VUS, variant of unknown significance; NA, not applicable; unavailable, lack of structural coverage or accuracy at nucleotide position.
